# Physiologically Based Pharmacokinetic Modeling of Tofacitinib: Predicting Drug Exposure and Optimizing Dosage in Special Populations and Drug–Drug Interaction Scenarios

**DOI:** 10.3390/ph18030425

**Published:** 2025-03-18

**Authors:** Zhihai Cao, Zilong Wang, Qian Zhang, Wei Zhang, Liang Zheng, Wei Hu

**Affiliations:** 1 Department of Clinical Pharmacology, The Second Affiliated Hospital of Anhui Medical University, Hefei 230601, China; caozh0826@163.com (Z.C.); zilongwang013@gmail.com (Z.W.); zhangqian@ahmu.edu.cn (Q.Z.); zhangpharmacy@163.com (W.Z.); 2 School of Pharmacy, Anhui Medical University, Hefei 230032, China

**Keywords:** PBPK, tofacitinib, drug–drug interaction, pediatrics, hepatic impairment, renal impairment

## Abstract

**Background:** Tofacitinib is mainly used in the adult population for immune-mediated inflammatory diseases. There is little information available on the pharmacokinetics of tofacitinib in pediatric patients, populations with hepatic impairment and renal impairment, and patients with drug–drug interactions (DDIs). This study aimed to develop a physiologically based pharmacokinetic (PBPK) model to predict the pharmacokinetics of tofacitinib in the populations mentioned above. **Methods:** We developed the PBPK models in PK-Sim^®^ and evaluated the models with observed clinical PK data. The Monte Carlo algorithm was used for parameter identification. **Results:** The adult PBPK model accurately simulated the pharmacokinetic profiles of all administration scenarios. The geometric mean fold errors for the predicted/observed maximum concentration and area under the curve are 1.17 and 1.16, respectively. The extrapolated models accurately simulated the pharmacokinetic characteristics of tofacitinib. The pediatric patients aged 12-to-<18 years and 2-to-<6 years need to adjust the dose to 4 mg BID and 1.7 mg BID, respectively, to achieve comparable steady-state exposures to 5 mg BID in adults. The populations with moderate hepatic impairment and severe renal impairment need to reduce the dose to 50% and 75% of the original dose, respectively. Tofacitinib should be reduced to 50% and 65% of the original dose for concomitant use with fluconazole and ketoconazole, respectively, and increased to 150% of the original dose for concomitant use with rifampicin. **Conclusions:** We developed a tofacitinib PBPK model and extrapolated it to special populations and DDIs. The predictive results of the models can help the rational use of tofacitinib in these populations.

## 1. Introduction

Tofacitinib is a potent oral small-molecule Janus kinase (JAK) inhibitor that selectively inhibits JAK1 and 3, with lower activity on JAK2 and TYK2 [1]. JAK inhibitors belong to a new class of orally targeted disease-modifying antirheumatic drugs that have revolutionized the therapeutic landscape for rheumatoid arthritis (RA) and other immune-mediated inflammatory diseases (IMIDs) [2]. Tofacitinib was the first JAK inhibitor approved for the treatment of RA and has since gained approval for the treatment of other IMIDs such as psoriatic arthritis, Crohn’s disease, and ulcerative colitis [3,4]. Tofacitinib monotherapy and combination therapy with conventional drugs is effective in reducing signs and symptoms of the disease and improving health-related quality of life, with benefits continuing over the long-term treatment period [5]. Currently, tofacitinib is primarily administered orally in clinical practice, with the most common formulations being oral solutions and tablets. The tablet formulations include conventional tablets, immediate-release (IR) tablets, and extended-release (XR) tablets, catering to different therapeutic requirements [6,7]. The standard recommended dosage of tofacitinib is 5 mg twice daily, and it is available in multiple formulations, including a 1 mg/mL oral solution, 5 mg IR tablets taken twice daily, and 11 mg XR tablets administered once daily [8,9].

The pharmacokinetics of tofacitinib exhibited linear and dose-proportional characteristics across a range of doses, from 0.1 mg to 100 mg [10]. Tofacitinib is rapidly well absorbed in the gastrointestinal tract with an oral absolute bioavailability of 74% in healthy individuals [11]. Tofacitinib primarily binds to albumin with an unbound fraction of 61% in plasma [12]. Tofacitinib is mainly metabolized by cytochrome P450 (CYP) 3A4 and CYP2C19 with metabolic contributions of 53% and 17%, respectively; the remaining 30% are excreted in urine after renal filtration and tubular secretion of the parent drug [11,13].

Since tofacitinib is metabolized by CYP3A4 and CYP2C19, the concomitant administration of drugs that affect the activity of these two enzymes will have an effect on the pharmacokinetics (PKs) of tofacitinib [14]. In a clinical trial [15], the integration of tofacitinib with fluconazole led to a significant enhancement in its pharmacokinetic parameters, with the area under the curve (AUC) increasing by 79% and the peak plasma concentration (C_max_) rising by 27%. Similarly, when tofacitinib was co-administered with ketoconazole, the AUC surged by 103% and the C_max_ increased by 16% [15]. Additionally, the half-life of tofacitinib was extended by roughly one hour in the presence of either fluconazole or ketoconazole [15]. In a separate clinical trial [16], co-administration of tofacitinib with rifampicin, a CYP3A4 and CYP2C19 inducer, resulted in a significant reduction in systemic exposure. The AUC and C_max_ of tofacitinib decreased to 16% and 26% of the baseline values, respectively [16]. These findings indicate that concomitant administration of agents that modulate the activity of tofacitinib-metabolizing enzymes may significantly alter systemic exposure to tofacitinib. Consequently, such drug interactions warrant careful clinical evaluation, including potential dose modifications or therapeutic restrictions, to ensure optimal efficacy and minimize the risk of adverse events.

Tofacitinib has received regulatory approval for the treatment of juvenile idiopathic arthritis (JIA) and juvenile psoriatic arthritis (JPsA) [17]. However, its clinical application in pediatric populations currently relies predominantly on empirical dosing strategies [18]. Conventional pediatric dosing approaches based solely on body weight or age fail to adequately account for organ-specific developmental changes and clearance maturation processes [19]. Ongoing clinical trials are specifically examining the use of tofacitinib in children to address these considerations [20]. In a previously published clinical trial [21] of tofacitinib in children with JIA, tofacitinib is administered in either tablet or oral solution formulations, with the selection of dosage form and strength guided by the patient’s age and body weight. PK analyses revealed a comparable median time to peak plasma concentration (T_max_) across all study cohorts, revealing an age-dependent decrease in both apparent clearance and volume of distribution [21]. The pediatric PK findings served as the foundation for establishing the optimized dosing regimen implemented in subsequent clinical trials within the tofacitinib pediatric development program [21].

The liver plays a central role in drug metabolism, and hepatic impairment can significantly impact pharmacokinetic processes by reducing the clearance of both hepatically metabolized drugs and those undergoing biliary excretion. This effect exhibits a severity-dependent relationship, with more pronounced alterations observed in patients with moderate-to-severe hepatic dysfunction [22,23]. Similarly, renal impairment affects the elimination of renally excreted drugs, potentially leading to systemic drug accumulation and an elevated risk of toxicity. The extent of these pharmacokinetic disruptions is directly linked to the level of renal impairment, with more profound dysfunction leading to more significant pharmacokinetic changes [24,25]. Two published clinical studies [26,27] have systematically evaluated alterations in the pharmacokinetic profiles of tofacitinib following oral administration in subjects with varying degrees of hepatic and renal impairment, with comparative analyses performed against matched healthy controls, respectively. In mild hepatic impairment, tofacitinib’s AUC from 0 to infinity (AUC_inf_) and C_max_ remained comparable to healthy volunteers [26]. However, moderate impairment resulted in 65% and 49% increases in the AUC_inf_ and C_max_, respectively [26]. Patients with renal impairment showed an increased AUC_inf_ compared to those with normal function: there was mild impairment at 137%, moderate impairment at 143%, and severe impairment at 223% [27]. The C_max_ remained consistent across groups, while t_1/2_ was prolonged with worsening renal damage [27]. Hence, it is imperative for clinicians to modify drug dosages in alignment with the severity of organ dysfunction to guarantee the potency of the treatment and to reduce the risk of adverse effects in patients with hepatic and renal insufficiencies.

PK data are essential for evaluating the safety and efficacy of drugs. Physiologically based pharmacokinetic (PBPK) modeling can simulate the in vivo process of drugs based on anatomical physiology and drug properties, providing PK insights into populations for whom empirical data are lacking, including DDIs, pediatrics, and hepatic and renal impairment populations. The objective of this study was to develop a PBPK model for the general adult population and then extrapolate it to special populations and DDIs, thereby predicting the pharmacokinetics of tofacitinib in DDIs, pediatrics, and hepatic and renal impairment populations.

## 2. Results

### 2.1. Tofacitinib Base Model Development and Validation

**Figure 1:**
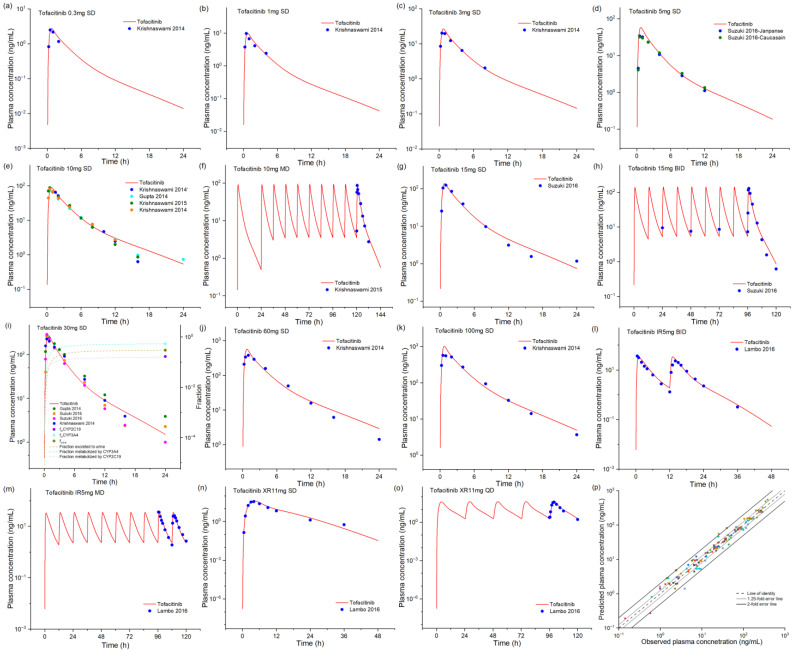
The observed and model-predicted plasma concentration–time profiles of tofacitinib following oral administration (**a**–**o**) [10,15,27,28,29,30]. The red lines indicate median concentration data from model predictions and blue dots indicate reported concentrations from different studies. The goodness-of-fit plot for model-predicted tofacitinib concentrations (**p**). Different colors represent data from different simulations. Black and gray trend lines indicate 2-fold and 1.25-fold prediction errors, respectively. IR, immediate release. XR, extended release.

We developed and validated a comprehensive PBPK model for oral tofacitinib in adults, encompassing both single and multiple dosing regimens across a wide dose range (0.3–100 mg) for plain tablets and IR and XR formulations. The model was validated against published clinical pharmacokinetic data, demonstrating robust predictive performance. As shown in Figure 1, the model accurately captured the plasma concentration–time profiles across all dosing scenarios. Mechanistically, the model incorporated physiologically realistic contributions of metabolic enzyme clearance pathways and renal excretion, which were consistent with reported values in the literature. The model’s predictive accuracy was quantitatively assessed through comparison of key pharmacokinetic parameters (C_max_, AUC, and T_max_) between simulated and observed data, as detailed in Supplementary Table S1. The fold errors (FEs) of the predicted/observed pharmacokinetic parameters C_max_, AUC, and T_max_ are within the range of 0.79–1.64, 0.85–1.45, and 0.75–1.50, respectively. One AUC and one T_max_ value exceeded the above range. The geometric mean fold errors (GMFEs) further confirmed model reliability, with values of 1.17 (C_max_), 1.16 (AUC), and 1.27 (T_max_) across all simulated studies. The predictive performance was further validated through goodness-of-fit analysis, as shown in Figure 1p. The plot revealed that 97% of predicted plasma concentrations fell within a 2-fold error range of observed values, with 79% falling within a more stringent 1.25-fold error range, demonstrating the model’s high precision and reliability for pharmacokinetic predictions. According to the sensitivity analysis (Figure S1), tofacitinib exposure is sensitive to the plasma unbound fraction, liphophilicity, specific intestinal permeability, and CYP3A4 metabolism kinetics, apart from formulation-related parameters.

### 2.2. PBPK Model in Pediatric Patients

**Figure 2:**
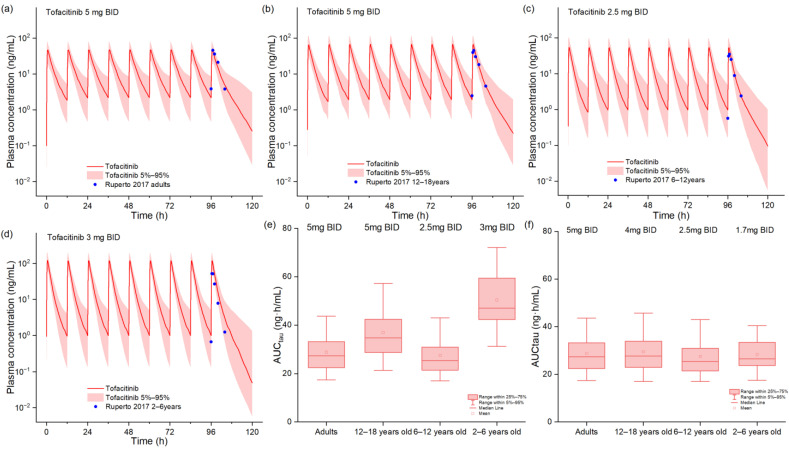
The observed and model-predicted plasma concentration–time profiles of tofacitinib in pediatric patients (**a**–**d**) [21]. The red lines indicate median concentration data from model predictions, while the shaded areas indicate a 5%-to-95% concentration range, and blue dots indicate reported concentrations from different studies. Box–whisker plots of pediatric patients at different cohorts after oral tofacitinib to compare AUC values (**e**,**f**).

The predictive performance of the pediatric PBPK model is illustrated in Figure 2, which compares simulated and observed plasma concentration–time profiles across different age groups. The figure effectively demonstrates that the pediatric model for tofacitinib accurately predicted plasma concentrations of tofacitinib in pediatric patients aged 12-to-<18 years (Cohort 1) and 6-to-<12 years (Cohort 2), as well as in adults as the reference. However, the model exhibited an overprediction of plasma concentrations in younger children aged 2-to-<6 years (Cohort 3), suggesting potential limitations in physiological parameterization for this age group. Quantitative analysis of model performance, as presented in Table S2, revealed that all FEs for the C_max_ and AUC across the three pediatric cohorts remained within the acceptable two-fold range, confirming the model’s overall validity for dose extrapolation. Based on exposure-matching analysis (Figure 2e,f), we established age-stratified dosing recommendations: while Cohort 2 could maintain a 2.5 mg BID regimen, dose adjustments were recommended for other groups to achieve therapeutic exposures equivalent to adult 5 mg BID dosing. Specifically, Cohort 1 required a reduction to 4 mg BID, while Cohort 3 needed a lower dose of 1.7 mg BID to compensate for the observed pharmacokinetic differences.

### 2.3. PBPK Model in Hepatic Impairment Populations

**Figure 3:**
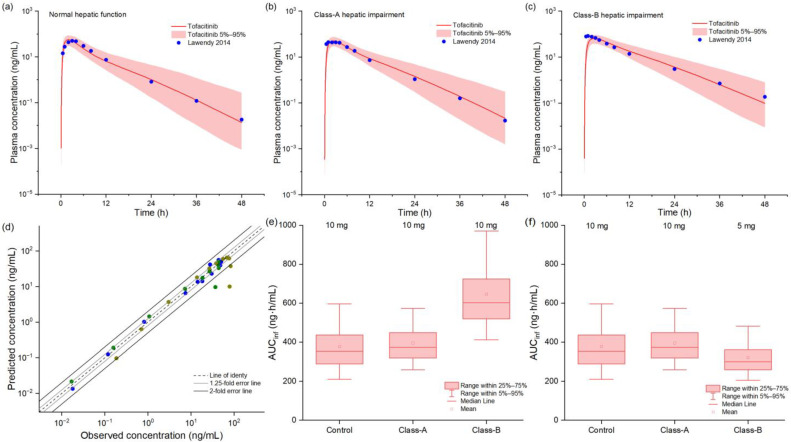
The observed and model-predicted plasma concentration–time profiles of tofacitinib in populations with hepatic impairment (**a**–**c**) [26]. The red lines indicate median concentration data from model predictions, while the shaded areas indicate a 5%-to-95% concentration range, and blue dots indicate reported concentrations from different studies. The goodness-of-fit plot for model-predicted tofacitinib concentrations (**d**). Different colors represent data from different simulations. Black and gray trend lines indicate 2-fold and 1.25-fold prediction errors, respectively. Box–whisker plots of populations with different hepatic functions after oral tofacitinib to compare AUC values (**e**,**f**).

The PBPK model demonstrated robust predictive performance across various stages of hepatic impairment (HI), as evidenced by the close agreement between simulated and observed pharmacokinetic profiles (Figure 3). Goodness-of-fit analysis revealed that 91% of predicted tofacitinib concentrations fell within a 2-fold error range of observed values, with 85% falling within a more stringent 1.25-fold error range, indicating excellent model accuracy. Quantitative validation results, presented in Table S3, showed that FEs for predicted versus observed C_max_ and AUC values consistently fell within acceptable ranges, confirming the model’s reliability for hepatic impairment simulations. Based on exposure-matching analysis using box–whisker plots, we established distinct dosing recommendations: while patients with mild HI could maintain original dosing regimens, those with moderate HI required a 50% dose reduction to achieve comparable drug exposure to healthy subjects, thereby maintaining therapeutic efficacy while minimizing potential toxicity risks.

### 2.4. PBPK Model in Renal Impairment Populations

**Figure 4:**
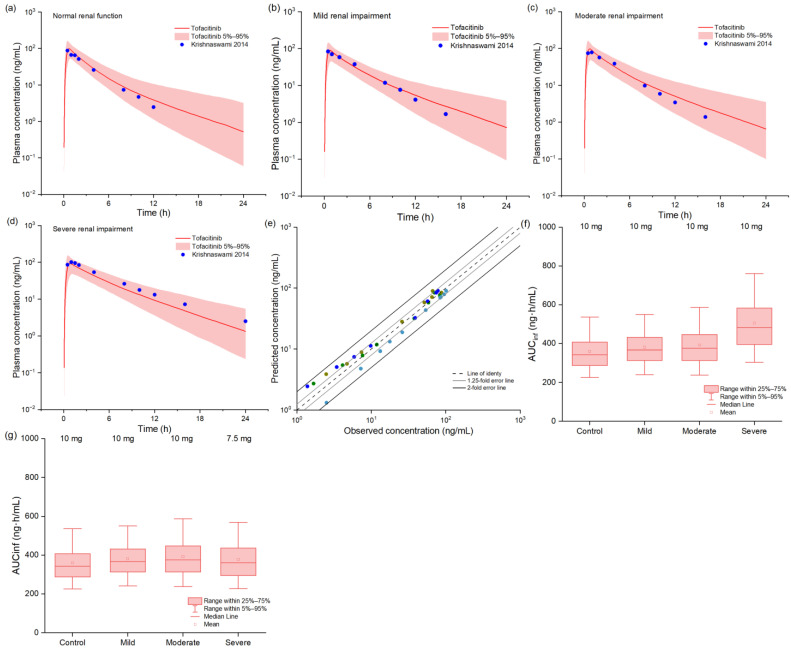
The observed and model-predicted plasma concentration–time profiles of tofacitinib in populations with renal impairment (**a**–**d**) [27]. The red lines indicate median concentration data from model predictions, while the shaded areas indicate a 5%-to-95% concentration range, and blue dots indicate reported concentrations from different studies. The goodness-of-fit plot for model-predicted tofacitinib concentrations (**e**). Different colors represent data from different simulations. Black and gray trend lines indicate 2-fold and 1.25-fold prediction errors, respectively. Box–whisker plots of populations with different renal functions after oral tofacitinib to compare AUC values (**f**,**g**).

Figure 4 presents the comparative analysis between observed and predicted systemic concentrations of tofacitinib following a 10 mg oral dose in healthy adults and patients across the spectrum of renal impairment (RI), from mild to severe stages. The model demonstrated excellent predictive accuracy, with all simulated concentrations falling within a 2-fold error range of observed values and approximately 70% within a more stringent 1.25-fold error range, as evidenced by goodness-of-fit analysis. Quantitative validation results, detailed in Table S3, confirmed the model’s reliability, with FEs for predicted versus observed C_max_ and AUC values consistently maintained between 0.5 and 2.0 across all renal function groups. Based on comprehensive box–whisker analysis, we established renal function-dependent dosing recommendations: while patients with mild-to-moderate RI could maintain standard dosing regimens, those with severe RI required a 25% dose reduction to achieve therapeutic exposures comparable to those in healthy individuals, thereby optimizing the risk–benefit ratio in this vulnerable population.

### 2.5. Drug–Drug Interactions Model of Tofacitinib with Perpetrators

**Figure 5:**
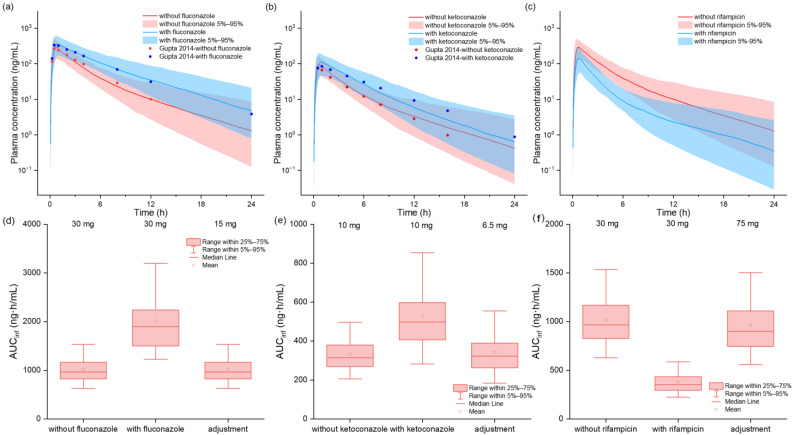
The observed and model-predicted plasma concentration–time profiles of tofacitinib with fluconazole, ketoconazole, and rifampicin (**a**–**c**) [15]. The red and blue lines indicate median concentration data from model predictions in the absence and presence of perpetrators, while the shaded areas indicate a 5%-to-95% concentration range, and solid dots indicate reported concentrations from different studies. Box–whisker plots of populations in the absence and presence of perpetrators after oral tofacitinib to compare AUC values (**d**–**f**).

As demonstrated in Figure 5a,b, the developed PBPK models successfully predicted the DDI profiles of tofacitinib when co-administered with fluconazole and ketoconazole, showing excellent agreement with observed clinical data. For the fluconazole interaction, the model predicted the C_max_ ratio (C_max_R) of 1.33 and AUC ratio (AUCR) of 1.89, closely matching the observed values of 1.27 (95% CI: 1.12–1.44) and 1.75 (95% CI: 1.64–1.96), respectively. Similarly, for ketoconazole co-administration the predicted C_max_R of 1.26 and AUCR of 1.56 aligned well with the measured values of 1.15 (95% CI: 1.05–1.29) and 2.04 (95% CI: 1.91–2.16). In the case of rifampicin co-administration, the model predicted a C_max_R of 0.52 and AUCR of 0.37, compared to the observed values of 0.26 (95% CI: 0.23–0.31) and 0.16 (95% CI: 0.14–0.18), respectively, as summarized in Table 1. Based on these DDI predictions, we established the following dose adjustment recommendations to maintain therapeutic exposures equivalent to those achieved in the absence of interacting drugs: (1) a 50% dose reduction when co-administered with fluconazole, (2) a 35% dose reduction with ketoconazole, and (3) a 150% dose increase with rifampicin.

## 3. Discussion

The PBPK model has evolved into an effective tool for providing targeted guidance by simulating and extending clinical trial results in individuals being treated with DDIs and special populations, including pediatrics and those with hepatic or renal impairment. This advanced approach optimally harnesses existing PK data to inform precise and personalized therapeutic strategies [31,32,33]. In the absence of sufficient clinical data, the PBPK model is an effective tool for predicting PK profiles in special populations and DDIs, thus helping to rationalize medication decisions. We were the first to develop the PBPK model of tofacitinib using PK-Sim^®^ and extrapolate it to DDIs and special populations such as pediatric patients and patients with hepatic or renal impairment. These models could provide a basis for dose selection and adjustment for the clinical use of tofacitinib in special populations and DDIs. With the validation of limited clinical data, our models achieved acceptable results and are expected to provide a scientific basis for the rational use of medication in these populations. The base PBPK model of tofacitinib accurately predicted the plasma concentration of tofacitinib in healthy adults and the contribution of CYP3A4 and CYP2C19 to clearance and the proportionof renal excretion were 53%, 16%, and 29%, respectively, which were consistent with data reported in the literature [11,14]. By extrapolating the adult PBPK model to special populations and DDIs, comparing predicted and observed plasma concentration–time curves and calculating FEs for the AUC and C_max_ or the AUCR and C_max_R demonstrated the reliability of the models we developed. In addition, assuming that the area under the curve in the dosing interval (AUC_tau_) values and AUC_inf_ values are similarly characterized in adults, special populations, and DDIs, we recommend the dose selection and adjustment for these populations.

The developed pediatric PBPK model for tofacitinib demonstrated strong predictive performance when validated against clinical observed data, successfully capturing plasma concentration profiles in both Cohort 1 and Cohort 2. However, the model exhibited an overestimation of PK characteristics in Cohort 3. This discrepancy may be attributed to specific modifications implemented during the clinical trial design. Although Cohort 3 had a significantly lower median body weight compared to Cohort 1 and Cohort 2, the clinical trial investigators implemented a protocol amendment following interim PK analysis of Cohort 1 and Cohort 2. Specifically, based on emerging PK data from Cohort 1 and Cohort 2, the dosing regimen for Cohort 3 was adjusted upward to achieve comparable systemic exposure, resulting in an increase in the administered dose relative to the initial weight-based calculation. This protocol adjustment, while clinically justified, may have contributed to the observed model overprediction in the youngest cohort, as the PBPK model was initially parameterized using standard weight-based scaling approaches without accounting for this specific clinical trial adaptation. Furthermore, we hypothesize that the observed model deviation may also reflect physiological immaturity in children aged 2-to-<6 years, particularly in drug-metabolizing organs and systems. This population exhibits ongoing development of hepatic enzyme systems (particularly CYP3A4 and CYP2C19), renal function maturation, and gastrointestinal tract development, all of which significantly influence tofacitinib’s PK profile. The current model may not fully capture these developmental pharmacokinetic changes, as it was primarily developed and validated using data from more mature pediatric populations. Additional factors such as age-dependent changes in body composition (e.g., higher body water content, lower muscle mass) and plasma protein binding capacity may further contribute to the observed discrepancies. These findings highlight the need for more comprehensive characterization of developmental pharmacology in younger pediatric populations to improve PBPK model predictability across all age groups.

Fluconazole and ketoconazole, known as CYP3A4 inhibitors, exhibit distinct inhibitory potencies, with fluconazole acting as a moderate CYP3A4 inhibitor and potent CYP2C19 inhibitor and ketoconazole serving as a potent CYP3A4 inhibitor [34,35]. Leveraging available clinical data, we developed DDI models to characterize the pharmacokinetic interactions between tofacitinib and these commonly used CYP inhibitors. The model successfully simulated the plasma concentration profiles, demonstrating a particular accuracy in predicting both the C_max_R and AUCR. The model predictions for these parameters fell within the 90% confidence interval of the observed mean values, indicating robust predictive performance. Similarly, we developed a DDI model to characterize the interaction between tofacitinib and rifampicin, a potent CYP3A4 inducer and moderate CYP2C19 inducer. However, constrained by limited clinical data for comparative plasma concentration profiles, the model predictions for both the C_max_R and AUCR exceeded the clinically observed values. This discrepancy aligns with findings reported in previously published literature [36] and may be attributed to several mechanistic limitations in the current rifampicin model. Specifically, the model’s representation of rifampicin’s induction mechanisms may not fully capture the complex interplay between CYP3A4 and CYP2C19 induction and tofacitinib’s metabolic pathways. Additionally, potential time-dependent effects of rifampicin’s induction capacity and its impact on hepatic enzyme turnover rates may not be adequately incorporated in the current model structure.

The clinical study in hepatic impairment populations of tofacitinib was conducted under postprandial conditions, reflecting the well-documented food effect on PK characteristics of tofacitinib. To account for this critical physiological variable, we adjusted the formulation release of tofacitinib into our model. The optimized model demonstrated excellent predictive performance, with simulated plasma concentration–time profiles showing strong concordance with observed clinical data. Notably, the model successfully characterized the PK profiles of orally administered tofacitinib across varying degrees of hepatic impairment, from mild to moderate impairment. Similarly, our PBPK modeling approach demonstrated robust predictive performance in characterizing the pharmacokinetics of tofacitinib across various stages of renal impairment. The model successfully simulated the drug’s PK profile in patients with mild, moderate, and severe renal impairment, achieving excellent agreement with observed clinical data. This comprehensive characterization of tofacitinib’s renal impairment profile provides valuable insights into dose adjustment strategies in this special population. Based on comprehensive analysis of available clinical studies and our modeling results, we have developed preliminary dosage recommendations for special populations. These models enable quantitative prediction of exposure–response relationships in these special populations, providing a scientifically robust basis for dose adjustment recommendations. The model’s performance suggests its potential utility in optimizing dosing regimens for hepatic and renal impairment patients and supporting regulatory submissions for label recommendations guiding clinical trial design in special populations. While the current model demonstrates a promising predictive capability, its reliability can be further enhanced through validation with additional clinical datasets. With continued refinement and expanded validation, this model has the potential to serve as a valuable tool for dose optimization in hepatic impairment populations, ultimately supporting the safer and more effective use of tofacitinib in these clinically challenging scenarios.

The PBPK model of tofacitinib has previously been developed and validated in Simcyp^®^ [36]. This investigation employed a PBPK modeling approach to systematically evaluate and predict the impact of drug–drug interactions and varying degrees of hepatic or renal impairment on tofacitinib’s pharmacokinetic profiles. In contrast to the previous investigation, our current study incorporated a more comprehensive dataset for model validation and extended the simulation scope to include both single and multiple dosing regimens, encompassing immediate-release formulations along with their extended-release counterparts. In light of the limited data pertaining to intravenous drug administration and its restricted clinical application, we chose not to develop a corresponding model. The predicted/observed ratios of pharmacokinetic parameters in both hepatic and renal impairment models demonstrated a significantly improved model fitting accuracy compared to the previous study. We also offer adjustments for dosages in the event of drug interactions. But a consistent underprediction of the DDI extent between tofacitinib and rifampicin was observed in both PBPK models, suggesting potential limitations in the current modeling approach. Moreover, we undertook a PBPK model in pediatric patients for tofacitinib, which exhibited strong predictive performance. This modeling effort yielded targeted dosage recommendations for children across distinct age categories, designed to align their drug exposures with those observed in adults.

The limitations of this study are as follows: (1) Model validation in special populations, including pediatric, hepatic impairment, and renal impairment groups, was restricted to single clinical studies for each population, potentially limiting the generalizability of the findings. (2) While the model incorporated well-characterized CYP-mediated drug interactions with perpetrators such as fluconazole (CYP2C19/CYP3A4 inhibitor), ketoconazole (potent CYP3A4 inhibitor), and rifampicin (CYP3A4/CYP2C19 inducer), numerous other clinically relevant CYP perpetrators that may be co-administered with tofacitinib remain uninvestigated. The development of corresponding DDI models for these additional interactions is currently constrained by the absence of sufficient clinical data in the published literature. Future research directions should focus on addressing these data gaps through comprehensive clinical studies and systematic data collection, which will enable refinement of the current model and enhance its predictive accuracy across diverse clinical scenarios.

## 4. Materials and Methods

### 4.1. PBPK Modeling Platform and Related Software

PBPK modeling was performed using the Open Systems Pharmacology (OSP) Suite version 11.3 (PK-Sim^®^ & Mobi^®^, Bayer AG, Leverkusen, Germany, http://www.open-systems-pharmacology.org/ (accessed on 23 April 2024), available as freeware under the GPLv2 license). Parameter identification was performed using the Monte Carlo algorithm within PK-Sim^®^. The observed plasma concentrations from previous clinical studies were extracted by WebPlotDigitizer version 4.4 (Ankit Rohatgi, Austin, TX, USA) using published concentration–time profiles.

### 4.2. PBPK Base Model Development and Evaluation of Tofacitinib

To facilitate the application of tofacitinib in special populations and DDIs, we initially developed a base PBPK model for tofacitinib in the adult population. The physicochemical parameters, such as logP, fraction unbound in plasma, molecular weight, pKa, and solubility, were sourced from the scientific literature and the FDA’s pharmacology review. Partition coefficients were estimated using the Rodgers and Rowland method, while cellular permeabilities were determined using the PK-Sim standard. The in vitro clearance pathways for tofacitinib encompassed CYP3A4, CYP2C19, and renal clearance of the parent drug. Metabolic processes accounted for 70% of the total clearance of tofacitinib, with CYP3A4 and CYP2C19 contributing approximately 53% and 17% to the metabolic clearance, respectively [14,37]. The remaining 30% of tofacitinib was excreted in the urine as the unchanged parent compound, primarily via glomerular filtration and tubular secretion [11]. The use of Michaelis–Menten kinetics for CYP3A4-mediated metabolism and first-order kinetics for CYP2C19-mediated metabolism is based on the distinct enzymatic characteristics and metabolic behaviors of these CYP450 isoforms [38,39,40]. The Michaelis–Menten constant (K_m_) values were derived from the scientific literature [41]. The catalytic rate constant (K_cat_), which reflects the clearance by CYP3A4 and the specific clearance by CYP2C19, was optimized based on clinically reported tofacitinib blood concentration data. Tofacitinib is marketed in three distinct tablet formulations: regular, IR, and XR. For the regular tablets, we assumed immediate dissolution, while for the IR and XR formulations we optimized the release parameters, including the 50% dissolution time and dissolution shape of the Weibull equation. Additionally, we refined the transcellular intestinal permeability of tofacitinib. The comprehensive input compound parameters for tofacitinib are detailed in Table 2.

We constructed a virtual White American individual, aged 30, to represent the average characteristics within the population. To assess the model’s predictive accuracy, we conducted population simulations using a cohort of 200 virtual Americans (50% female), spanning ages 18 to 60. The observed data references, along with the dosing regimen and formulation details, are provided in Table S4. The FE and GMFE were employed to quantify the discrepancies between predicted and observed PK parameter values, thereby assessing the precision of the PBPK models [43]. The consensus among most model developers is that for a model to be deemed acceptable and to ensure sufficient predictive performance, the predicted PK parameters should fall within a range of 0.5-to-2-times the corresponding observed values [44]. The relevant equations are presented in Equations (1) and (2).

(1) $$FE=\frac{predicted\;PK\;parameter}{observed\;PK\;parameter}$$

(2) $$GMFE=10^{(\sum \left|\log\frac{predicted\;PK\;parameter}{observed\;PK\;parameter}\right|)/n}$$

In the above equation, n is the number of simulations.

The sensitivity of the final models to single parameters was assessed through local sensitivity analysis, quantified by evaluating the relative changes in the AUC. For single-dose administration drugs, this was measured as the change in the AUC_inf_, while for multiple-dose administration drugs the change in the AUC_tau_ was evaluated. The sensitivity is calculated using the following equation:

(3) $$Sensitivity=\frac{∆AUC}{AUC}\cdot \frac{p}{∆p}$$

where ΔAUC represents the change in the AUC, AUC represents the original model AUC value, Δp represents the change in the examined model parameter value, and p represents the original model parameter value. A sensitivity value of +1.0 denotes that a 10% increase in the examined parameter causes a 10% increase in the simulated AUC.

### 4.3. Extrapolation to Pediatric Patients

Having developed the tofacitinib foundational PBPK model, we leveraged the age-dependent algorithms integrated within PK-Sim^®^ for pediatric extrapolation. We refined anatomical and physiological parameters, encompassing body weight, height, organ volumes and ratios, blood flow, hematocrit levels, total body water content, as well as lipid and protein concentrations, among other factors. Following this, we applied scaling to the unbound fraction of tofacitinib, its metabolic processes, and renal clearance. To assess the performance of the pediatric model, we extracted clinical study data on pediatric populations from the literature [21] and utilized adult dosing results from another source [45] as a benchmark. The pediatric patients were divided into three age-based cohorts: Cohort 1, encompassing individuals aged 12-to-<18 years; Cohort 2, aged 6-to-<12 years; and Cohort 3, aged 2-to-< 6 years. We then assembled corresponding populations, each comprising 100 individuals (with a 70% female representation). To validate the PBPK model’s efficacy in pediatric populations, we conducted simulations and compared the outcomes with the observed PK curves from the clinical study. We normalized the pediatric doses to the actual adult dose and aligned the exposure levels with those of the adult population.

### 4.4. Extrapolation to the Hepatic Impairment Populations

Utilizing the established base model, we extrapolated its application to a population with hepatic impairment. We fine-tuned physiological parameters, such as blood flow, and liver-related anatomical parameters to create virtual individuals with varying degrees of hepatic function. The subjects were categorized into groups with normal hepatic function, mild hepatic impairment (Child–Pugh A, Class A), and moderate hepatic impairment (Child–Pugh B, Class B), in accordance with the criteria from a clinical study [26]. Understanding that the absorption of drugs can be substantially affected by food intake, we optimized the dissolution parameters, including the time and shape of the Weibull equation, to account for postprandial administration. Subsequently, we independently constructed corresponding populations, each consisting of 1000 individuals (with a 25% female demographic). We conducted simulations of the concentration–time curves for healthy adults and for patients with Class A and Class B hepatic impairment. Following this, we normalized the doses for each population group to the actual dose administered to the healthy population, ensuring that the exposure levels matched those of the healthy cohort.

### 4.5. Extrapolation to the Renal Impairment Populations

Similarly, we extended the base model to accommodate a population with renal impairment. Beyond refining the previously adjusted parameters, we also tailored the estimated glomerular filtration rate (eGFR) to reflect renal function. Drawing from the findings of a clinical study [27], we classified the subjects into groups with normal renal function, mild renal impairment (RI), moderate RI, and severe RI, based on their eGFR values. We then set out to independently assemble the respective populations, each comprising 1000 individuals (with a 50% representation of females). Subsequent to this, we simulated the blood concentration–time curves for each of these distinct populations. Following the simulations, we normalized the doses for each group to match the actual dose administered to the healthy population, thereby ensuring that the exposure levels were consistent with those of the healthy cohort.

### 4.6. Drug–Drug Interactions Model of Tofacitinib with Fluconazole, Ketoconazole, and Rifampicin

Previously, PBPK models for fluconazole [46], ketoconazole [47], and rifampicin [48] have been published and validated. These established PBPK models were integrated with the tofacitinib PBPK model to create respective DDI models. The compound-specific input parameters for these PBPK models are detailed in Table S6. Subsequently, the models were employed to simulate the PK characteristics of tofacitinib, both with and without the co-administration of the interacting drugs, in accordance with the dosing regimens used in the clinical studies [15,36]. The predicted pharmacokinetic parameters were directly obtained from models’ outputs. To assess the PK of tofacitinib in the presence or absence of the interacting drugs, the AUCR and C_max_R were calculated by dividing the values obtained during co-medication by the corresponding control values [23]. Subsequently, dose normalizations were carried out for the population receiving co-administration with the perpetrators to match the actual dose administered to the non-perpetrator co-administration groups. The exposure levels were adjusted accordingly to ensure alignment with those of the non-perpetrator groups.

## 5. Conclusions

We built a tofacitinib PBPK model in adult populations and extrapolated it to DDIs and special populations, such as pediatric patients and patients with hepatic impairment or renal impairment. The models adequately capture the pharmacokinetic characteristics of tofacitinib, allowing us to predict the drug exposure in special populations and DDIs. These populations may require dosage adjustments to achieve an effective exposure, while concurrently maintaining vigilance regarding any potential effects on the populations. While the model provides an initial framework for PK prediction in these populations, further data are needed to validate the reliability of the model for dose recommendations.

## Figures and Tables

**Table 1:** Predicted and observed C_max_R and AUCR of tofacitinib in the presence of fluconazole, ketoconazole, and rifampicin.

Drugs	C_max_R		AUCR	
	Predicted/Mean	Observed/Mean(90%CI)	Predicted/Mean	Observed/Mean(90%CI)
Fluconazole	1.36	1.27 (1.12~1.44)	1.95	1.79 (1.64~1.96)
Ketoconazole	1.33	1.16 (1.05~1.29)	1.63	2.03 (1.91~2.16)
Rifampicin	0.52	0.26 (0.23~0.31)	0.37	0.16 (0.14~0.18)

**Table 2:** Input compound parameters for the tofacitinib PBPK model.

Parameters	Tofacitinib	Reference/Source
lipophilicity	1.15	[42]
plasma fraction unbound	0.61	[12]
MW (molecular weight)	312.40 g/mol	[12]
pKa	5.07	[12]
solubility	2.9 mg/mL	[12]
specific intestinal permeability	7.24 × 10^−6^ cm/min	optimized
partition coefficients calculation	diverse	Rodgers and Rowland
cellular permeability	4.37 × 10^−4^ cm/min	PK-Sim standard
formulation	normal dissolved, IR Weibull, XR Weibull, fed Weibull	
50% dissolution time	IR 0.29 h, XR 3.22 h, fed 1.25 h	optimized
dissolution shape	IR 0.59, XR 2.42, fed 1.01	optimized
GFR fraction	1.0	assumed
K_m,CYP3A4_	10.61 μmol/L	[41]
K_cat,CYP3A4_	0.49 L/min	optimized
f_m,CYP3A4_	0.53	[37]
CL_spec,CYP2C19_	0.06 L/min	optimized
f_m,CYP2C19_	0.17	[37]
TS_spec_	0.53 L/min	optimized
f_urine_	0.3	[37]

MW: molecular weight; pKa: acid dissociation constant; GFR: glomerular filtration; K_m_: Michaelis–Menten constant; k_cat_: V_max_ per recombinant enzyme; CL_spec_: specific clearance; IR: immediate release; XR: extended release; f_m_: fraction metabolized by a certain enzyme; TS_spec_: specific clearance of tubular secretion; f_urine_: fraction metabolized to urine.

## Data Availability

Data presented in this study are included in the article and Supplementary Material.

## Supplementary Materials

The following supporting information can be downloaded at https://www.mdpi.com/article/10.3390/ph18030425/s1, Table S1: Predicted and observed values for pharmacokinetic parameters of tofacitinib; Table S2: Predicted and observed values for pharmacokinetic parameters of tofacitinib in pediatrics; Table S3: Predicted and observed values for pharmacokinetic parameters of tofacitinib in hepatic and renal impairment populations; Table S4: Clinical pharmacokinetic reports used in tofacitinib base PBPK modeling; Table S5: Clinical pharmacokinetic reports used in tofacitinib PBPK modeling in special populations and DDIs; Table S6: Input compound parameters and inhibition/induction kinetics for the fluconazole, ketoconazole, and rifampicin PBPK models; Figure S1. The sensitivity analysis for tofacitinib PBPK model. Sensitivity of the final model was measured as the relative change of a specific pharmacokinetic parameter after a single dose of tofacitinib conventional tablets. A sensitivity value of +1.0 denotes that a 10% increase in the examined parameter causes a 10% increase in the pharmacokinetic parameter. The sensitivity values are presented as absolute values in figures.

## Abbreviations

The following abbreviations are used in this manuscript:

PK	Pharmacokinetics
DDI	Drug–drug interaction
PBPK	Physiologically based pharmacokinetic
CYP	Cytochrome P450
C_max_	Peak plasma concentration
AUC	Area under the curve
JAK	Janus kinase
RA	Rheumatoid arthritis
IMID	Immune-mediated inflammatory disease
IR	Immediate release
XR	Extended release
JIA	Juvenile idiopathic arthritis
JPsA	Juvenile psoriatic arthritis
T_max_	Time to peak plasma concentration
AUC_inf_	Area under the curve from 0 to infinity
FE	Fold error
GMFE	Geometric mean fold error
C_max_R	Peak plasma concentration ratio
AUCR	Area under the curve ratio
HI	Hepatic impairment
RI	Renal impairment
AUC_tau_	Area under the curve in the dosing interval
K_m_	Michaelis–Menten constant
K_cat_	Catalytic rate constant
eGFR	Estimated glomerular filtration rate
