# Supplementary material for: Physiologically Based Pharmacokinetic Modeling of Tofacitinib: Predicting Drug Exposure and Optimizing Dosage in Special Populations and Drug–Drug Interaction Scenarios
Source: Pharmaceuticals (Basel). 2025 Mar 18;18(3):425. doi: 10.3390/ph18030425 (PMC11945186; doi:10.3390/ph18030425)
Supplement: Supplementary file 1 [file pharmaceuticals-18-00425-s001.zip › pharmaceuticals-3521606-supplementary.pdf]

pharmaceuticals

MDPI

Supplementary Material

Table S1. Predicted and observed values for pharmacokinetic parameters of tofacitinib. Predicted values are shown as the median of population simulations.

Study	Protocols	Methods	C_{max}/$C_{max\text{-}ss}$ (ng/mL)	AUC/AUC_{ss} (ng·h/mL)	T_{max} (h)
Krishnaswami 2014	0.3 mg SD	Predicted	2.76	8.36	0.75
		Observed	2.65	3.91	0.50
		FE	1.04	2.14	1.50
	1 mg SD	Predicted	9.86	27.91	0.75
		Observed	10.50	19.20	0.50
		FE	0.94	1.45	1.50
	3 mg SD	Predicted	27.82	84.14	0.75
		Observed	21.80	69.50	0.50
		FE	1.28	1.21	1.50
	10 mg SD	Predicted	94.04	283.76	0.75
		Observed	88.00	283.00	0.50
		FE	1.07	1.00	1.50
	30 mg SD	Predicted	288.65	869.43	0.75
		Observed	240.00	933.00	0.50
		FE	1.20	0.93	1.50
	60 mg SD	Predicted	591.32	1780.63	0.75
		Observed	438.00	1710.00	1.00
		FE	1.35	1.04	0.75
	100 mg SD	Predicted	1011.41	3047.63	0.75
		Observed	638.00	2980.00	0.50
		FE	1.58	1.02	1.50
Suzuki 2016	5 mg SD Japanese	Predicted	57.40	151.66	/
		Observed	41.30	111.00	/
		FE	1.39	1.36	/
	5 mg SD Caucasian	Predicted	57.40	151.66	/
		Observed	34.90	119.00	/
		FE	1.64	1.27	/
	15 mg SD	Predicted	175.34	462.32	/
		Observed	141.00	399.00	/
		FE	1.24	1.16	/
	15 mg BID	Predicted	146.83	403.83	/
		Observed	136.00	445.00	/
		FE	1.07	0.91	/
	30 mg SD Japanese	Predicted	288.65	869.43	/
		Observed	315.00	754.00	/
		FE	0.91	1.15	/

Study	Protocols	Methods	$C_{max}/C_{max\text{-}ss}$ (ng/mL)	AUC/AUC_{ss} (ng·h/mL)	T_{max} (h)
	30 mg SD Caucasian	Predicted	288.65	869.43	/
		Observed	265.00	788.00	/
		FE	1.09	1.10	/
Gupta 2014	10 mg SD	Predicted	94.04	283.76	0.75
		Observed	81.00	244.00	0.50
		FE	1.16	1.16	1.50
	30 mg SD	Predicted	288.65	869.43	0.75
		Observed	289.00	1020.00	0.50
		FE	1.00	0.85	1.50
Krishnaswami 2015	10 mg SD	Predicted	94.04	283.76	0.75
		Observed	98.30	273.80	0.50
		FE	0.96	1.04	1.50
	10 mg MD	Predicted	97.21	283.76	0.75
		Observed	89.20	275.00	0.50
		FE	1.09	1.03	1.50
Krishnaswami 2014-renal impairment	10 mg SD	Predicted	94.04	283.76	0.75
		Observed	94.20	268.00	0.75
		FE	1.00	1.06	1.00
Lambo 2016	5 mg IR BID SD	Predicted	34.45	128.60	1.05
		Observed	41.20	120.30	0.50
		FE	0.84	1.07	2.10
	5 mg IR BID MD	Predicted	34.80	128.60	1.05
		Observed	44.10	132.80	1.00
		FE	0.79	0.97	1.05
	11 mg XR SD	Predicted	39.77	313.30	4.25
		Observed	36.70	246.50	4.00
		FE	1.08	1.27	1.06
	11 mg XR MD	Predicted	40.78	301.67	4.25
		Observed	38.70	272.90	4.00
		FE	1.05	1.11	1.06
		GMFE	1.17	1.16	1.27

IR: Immediate release; XR: eXtended release; SD: Singe dose; MD: multiple dose; BID: twice a day; FE: Fold error; GMFE: Geometric mean fold error.

Table S2. Predicted and observed values for pharmacokinetic parameters of tofacitinib in pediatrics.

Study	Cohort	Predicted Parameters		Observed Parameters		FE	
		C_{max} (ng/mL)	$AUC_{0\text{-}inf}$ (ng·h/mL)	C_{max} (ng/mL)	$AUC_{0\text{-}inf}$ (ng·h/mL)	C_{max}	$AUC_{0\text{-}inf}$
Ruperto 2017	12-18years 5mg BID	69.60	180.80	47.00	156.60	1.48	1.15
	6-12years 2.5mg BID	55.87	132.55	41.70	118.80	1.34	1.12
	2-6years 3mg BID	124.80	244.89	66.20	142.50	1.89	1.72

BID: twice a day; FE: Fold error.

Table S3. Predicted and observed values for pharmacokinetic parameters of tofacitinib in hepatic and renal impairment populations.

Disease	Population	Predicted Parameters		Observed Parameters		FE	
		C_{max} (ng/mL)	AUC_{0-inf} (ng·h/mL)	C_{max} (ng/mL)	AUC_{0-inf} (ng·h/mL)	C_{max}	AUC_{0-inf}
HI	HI control	62.00	362.30	56.28	354.10	1.10	1.02
	Mild HI	62.00	369.50	50.80	373.67	1.22	0.99
	Moderate HI	93.70	625.30	65.69	602.99	1.43	1.04
RI	RI control	94.20	268.00	102.44	342.55	0.92	0.78
	Mild RI	87.30	370.00	93.00	367.17	0.94	1.01
	Moderate RI	104.00	396.00	101.71	375.67	1.02	1.05
	Severe RI	111.00	615.00	96.29	483.38	1.15	1.27

HI: hepatic impairment; RI: renal impairment; FE: fold error.

Table S4. Clinical pharmacokinetic reports used in tofacitinib base PBPK modeling.

Study	Dosage	Ethnicity	Population	Age, mean(range)	Number of subjects	Females proportion
Gupta 2014	10 mg SD	European	Healthy	23-49	12	NR
	30 mg SD	American	Healthy	22-53	12	NR
Krishnaswami 2014-renal impairment	10 mg SD	American	Healthy	50.8(37-65)	6	NR
Krishnaswami 2014	0.3 mg SD	American	Healthy	33.5(23–44)	8	NR
	1 mg SD	American	Healthy	24.9(20–32)	8	NR
	3 mg SD	American	Healthy	29.1(21–39)	8	NR
	10 mg SD	American	Healthy	31.1(20–44)	8	NR
	30 mg SD	American	Healthy	29.4(20–41)	9	NR
	60 mg SD	American	Healthy	23.1(19–31)	8	NR
	100 mg SD	American	Healthy	24.7(19–30)	7	NR
Krishnaswami 2015	10 mg SD	Chinese	Healthy	25.7(22-35)	12	50%
	10 mg MD	Chinese	Healthy	25.7(22-35)	12	50%
Suzuki 2016	5 mg SD	Japanese	Healthy	34.1(24-44)	8	0
	5 mg SD	Caucasian	Healthy	38.0(25-52)	9	11%
	15 mg SD	Japanese	Healthy	34.1(24-44)	8	0
	15 mg BID	Japanese	Healthy	35.8(24-45)	8	38%
	30 mg SD	Japanese	Healthy	34.1(24-44)	8	0
	30 mg SD	Caucasian	Healthy	38.0(25-52)	9	11%
Lambo 2016	5 mg IR BID	European	Healthy	35.7(10.8)	24	0
	5 mg IR BID MD	European	Healthy	35.7(10.8)	24	0
	11 mg XR SD	European	Healthy	35.7(10.8)	24	0
	11 mg XR MD	European	Healthy	35.7(10.8)	24	0

SD: Singe dose; MD: multiple doses; BID: twice a day; IR: Immediate release; ER: Extended release; NR: Not reported.

Table S5. Clinical pharmacokinetic reports used in tofacitinib PBPK modeling in special populations and DDIs.

Study	Dosage	Ethnicity	Population	Age, mean(range)	Number of subjects	Females proportion
Ruperto 2017	5 mg BID	European	Healthy	52(12.8)	71	80.3%
	5 mg BID	European	Healthy	14.0(12.0-16.0)	8	62.5%
	2.5 mg BID	European	Healthy	10.0(8.0-11.0)	9	55.6%
	3 mg BID	European	Healthy	4.0(4.0-5.0)	9	77.8%
Lawendy 2014	10 mg SD	American	Healthy	53.5(51-58)	6	16.7%
	10 mg SD	American	Mild HI	53.3(36-64)	6	16.7%
	10 mg SD	American	Moderate HI	56.5(49-62)	6	16.7%
Krishnaswami 2014-renal impairment	10 mg SD	American	Healthy	50.8(37-65)	6	NR
	10 mg SD	American	Mild RI	61.2(57-65)	6	NR
	10 mg SD	American	Moderate RI	53.5(37-63)	6	NR
	10 mg SD	American	Severe RI	59.0(31-72)	6	NR
Gupta 2013	10 mg SD	European	Healthy	23-49	12	NR
	30 mg SD	American	Healthy	22-53	12	NR
Lamba 2012	30 mg SD	NR	Healthy	23-50	12	0

DDI: Drug-drug interaction; SD: Singe dose; BID: twice a day; RI: renal impairment; HI: hepatic impairment; NR: Not reported.

Table S6. Input compound parameters and inhibition/induction kinetics for the fluconazole, ketoconazole, and rifampicin PBPK models.

Parameters	Fluconazole	Ketoconazole	Rifampicin
Lipophilicity	0.83	2.52	2.50
plasma fraction unbound	0.89	0.01	0.17
MW	272.33 g/mol	487.43 g/mol	822.94 g/mol
pKa	2.03	6.51, 2.94	7.90, 1.70
solubility	6.90 mg/mL	variable	2.80 mg/L
partition coefficients calculation	Rodgers and Rowland	Berezhkovskiy	Rodgers and Rowland
cellular permeability	PK-Sim standard	PK-Sim standard	PK-Sim standard
GFR fraction	0.14	1.0	1.0
UGT2B7 specific clearance	1.85×10^{-3} 1/min	NA	NA
$K_{m,CYP3A4}$	NA	8.46×10^{-3} µmol/L	NA
$K_{cat,CYP3A4}$	NA	0.10 1/min	NA
$K_{m,AADAC}$	NA	1.88 µmol/L	195.10 µmol/L
$K_{cat,AADAC}$	NA	0.87 1/min	9.87 1/min
$K_{m,UGT1A4}$	NA	7.0 µmol/L	NA
$K_{cat,UGT1A4}$	NA	0.31 1/min	NA
$K_{i,CYP3A4}$	13.10 µmol/L	8.46×10^{-3} µmol/L	18.50 µmol/L
$K_{i,CYP2C19}$	2.0 µmol/L	NA	NA
$EC50_{CYP3A4}$	NA	NA	0.34 µmol/L
$Emax_{CYP3A4}$	NA	NA	9.0
$EC50_{CYP2C19}$	NA	NA	0.34 µmol/L
$Emax_{CYP2C19}$	NA	NA	4.23

MW: molecular weight; pKa: acid dissociation constant; GFR: glomerular filtration; Km: Michaelis-Menten constant; kcat: V_{max} per recombinant enzyme.

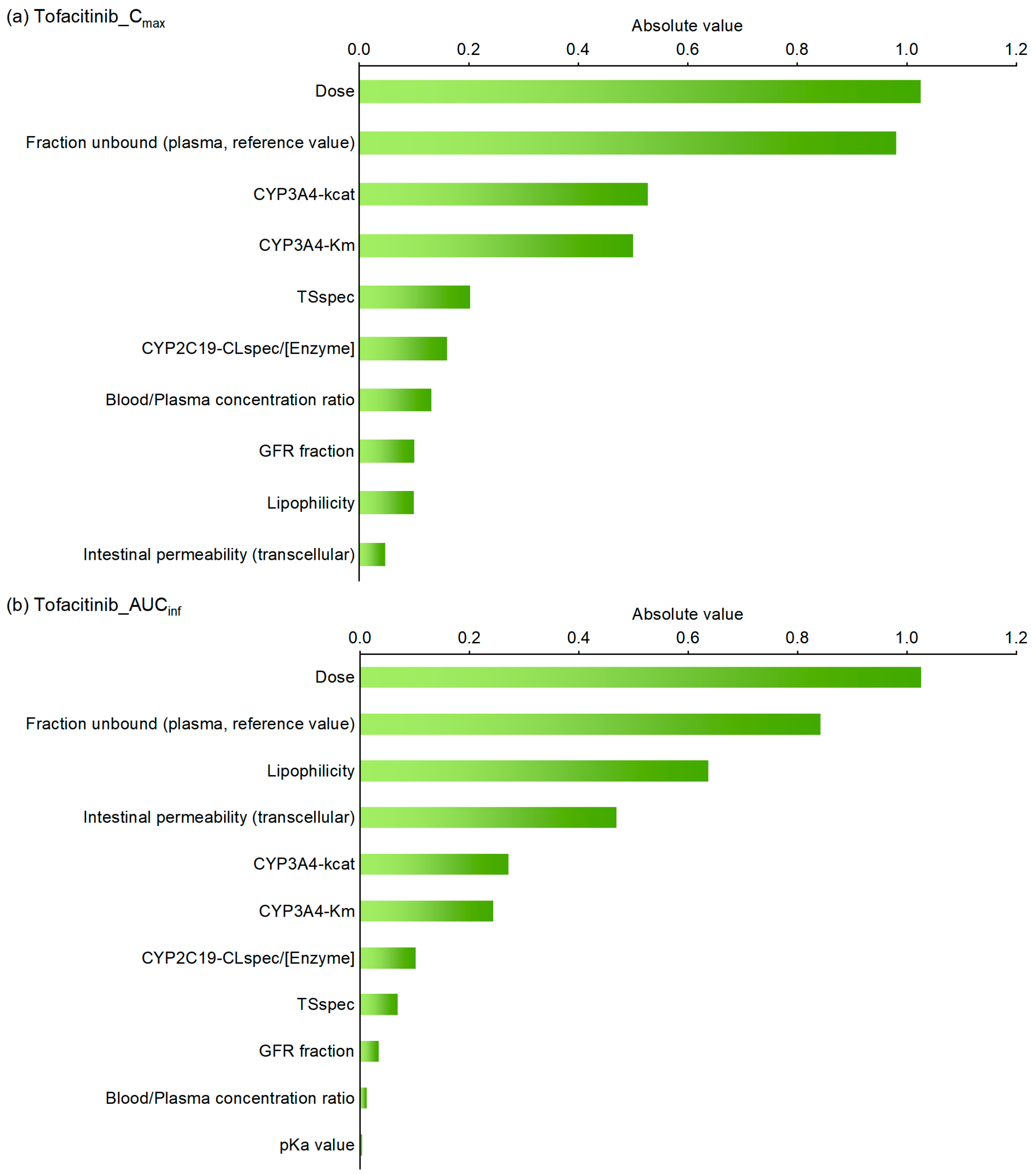

Figure S1. The sensitivity analysis for tofacitinib PBPK model. Sensitivity of the final model was measured as the relative change of a specific pharmacokinetic parameter after a single dose of tofacitinib conventional tablets. A sensitivity value of +1.0 denotes that a 10% increase in the examined parameter causes a 10% increase in the pharmacokinetic parameter. The sensitivity values are presented as absolute values in figures.
